# Instinct and the Unconscious
*Instinct and the Unconscious. By W. H. R. Rivers, M.D., D.Sc., LL.D., F.R.S. (Cambridge: University Press, 15s. net.)


**Published:** 1922-05

**Authors:** 


### THE REVIEW OF THE MONTH.

### INSTINCT AND THE UNCONSCIOUS.*

SIGMUND FREUD promulgated his epoch-making hypothesis of the unconscious mind just in time for the war to enable the truth of his theories to be tested on a large scale. And it was one of the few happy results of the war that it tempted from their academic seclusion men, such as Dr. Rivers, who, although members of the medical profession, had devoted themselves mainly to scientific investigations. Their intellects trained in the due weighing of evidence enabled them to judge controversial questions more fairly than others, perhaps, might have done. And of the work done by them this volume is a notable example.

In his sub-title, Dr. Rivers describes his book as "a contribution to a biological theory of the psycho-neuroses." He indicates that the special aim of the book is "to study the relation between instinct and that body of experience we are accustomed to speak of as the unconscious." It then becomes necessary to define certain terms. With this task Dr. Rivers commences.

The concept of the unconscious mind is revolutionary, and it is not surprising that the older psychologists found much difficulty in accepting it. Their aim had been to furnish a *rational* explanation of human conduct. In this attempt they failed, simply because much of human conduct is, as we now see, not rational, but instinctive. It is striking, as Dr. Rivers says, "that the due recognition of the importance of the unconscious and the first comprehensive attempt to formulate a scheme of its organisation and of the mechanisms by which it is brought into relation with the conscious should have come from those whose business it is to deal with the morbid aspect of the human mind. It is only the urgent and inevitable needs of the sick that have driven the physician into the full recognition of the unconscious."

The unconscious consists partly of experiences which are not capable of being brought into consciousness by any of the ordinary processes, and partly of tendencies derived from the great primitive instincts. In his insistence upon the former of these two great divisions of the contents of the unconscious Dr. Rivers, perhaps, a little neglects the latter. And in his desire, which is plainly expressed throughout the book, to avoid any suspicion of leaning to what he regards as Freud's undue insistence upon the sex instinct, the instinct of reproduction, he is inclined to regard the instinct of self-preservation as being more fundamental than that of sex. But it may well be argued, and it would be so argued by a Freudian, that all the activities of man, as of all other animals, are subservient to the one main object of the reproduction of the species. Michael Foster pointed this out many years ago. We possess the instinct of self-preservation, as well as all other instincts, in order that we may reproduce our species. And the unconscious may be said, in a sense, to regard all questions from this strictly animal point of view.

Dr. Rivers points out that the sceptical will be convinced of the reality of the unconscious if they will submit to the process of psycho-analysis, or (but less satisfactory) undertake a course of strict self-analysis. Dramatic experiences may be found lacking, but conviction of the reality of the unconscious will certainly be found. It is, in this connection, interesting to note that the educated laity has, on the whole, been more ready to accept the Freudian position than has the medical profession.

The passing of experience into the unconscious may occur at any age, and this fact has been strikingly illustrated by observations during the war. The obliteration may be due to mental shock or other physical factors. "Soldiers have lost the entire memory of their lives from some moment preceding a shock or severe strain until they have found themselves in hospital, perhaps weeks later, although during at least part of the intervening time they may have been to all appearance fully conscious, and may even have distinguished themselves by actions on the field of which they have no recollection."

How does experience become and remain unconscious? It has been customary to use the word "repression" for this process. But Dr. Rivers proposes the word "suppression" instead, and would reserve the former term for the process by which we wittingly endeavour to banish painful experience from consciousness. No doubt the two processes are distinct, if not fundamentally different. And there may be good reason for the use of the two terms. But a general agreement as to the sense in which terms are used is much to be desired, and confusion in this respect will tend to make an already sufficiently abstruse subject still more complicated and controversial.

One of the two processes just mentioned may lead on to the other. "Conscious repression seems often to lead to suppression. The suppression itself is unwitting, but the wish of the sufferer for suppression assists the process." Experience which tends to be forgotten or repressed is the immediately painful. Say that we forget an appointment in connection with which we anticipate painful emotions, the ultimate results may be even worse than the immediate experience from which we escape. But the process of active forgetting takes no heed of these ultimate consequences, it is directed to the avoidance of immediate discomfort. This is one instance of the childish character of the unconscious. Dr. Rivers sums up the matter in these words: "The special function of the unconscious is to act as a storehouse of instinctive reactions and tendencies, together with the experience associated with them, when they are out of harmony with the prevailing constituents of con-

* Instinct and the Unconscious. By W. H. R. Rivers, M.D., D.Sc., LL.D., F.R.S. (Cambridge: University Press, 15s. net.)

sciousness, so that, when present, they produce pain and discomfort."

An interesting account is given of the work of Head and his colleagues on the changes which accompany the healing of a severed cutaneous nerve. Two definite stages occur in this process. In the first, the protopathic stage, the sensations in the area innervated by the nerve are vague and crude, and tend to lead to movements which would withdraw the stimulated part from contact with the object to which the sensory changes are due. The second, the epicritic stage, is characterised by the return of normal cutaneous sensibility, and so makes possible the finer adjustments of behaviour. In the view of Dr. Rivers these two kinds of sensibility represent distinct stages in the development of the afferent nervous system. Certain manifestations of protopathic sensibility are suppressed as belonging to a crude form of nervous system, which has been superseded by a more efficient mechanism. The suppressed reactions have a strongly affective tone. They are "ready to spring into activity whenever the situation calls for an emotional rather than an intellectual response." From these considerations Dr. Rivers deduces the argument that "the suppression of conscious experience is only one example of a process which applies throughout the animal kingdom, and is essential to the proper regulation of every form of human or animal activity." "Every living process of the animal involves, not only activity devoted to the special end the animal has to meet, but also the inhibition of tendencies to activity of other kinds." "Certain elements of early experience are utilised and form, by fusion with other elements, the products which make up the experience of any later period of life. It is only elements of experience and modes of behaviour which are incompatible with these later developments which are suppressed." He would restrict the term "unconscious" to the earlier forms of mental experience which have not been utilised by the process of fusion. Much, however, of the contents of the unconscious is such on account of its having accompanied painful experience. And "experience which becomes unconscious through the agency of suppression either belong definitely to the affective aspect of mind, or, when intellectual in character, has been suppressed on account of its association with affective elements."

The nature of instinct must always be a question of much interest. We have to reject the old view that it is the mode of mental activity proper to animals as distinguished from the intelligence which was believed to be the main regulating factor in man's conduct. We now know that human conduct is far less subject to reason and intelligence than was once supposed. Especially is this so "in those social reactions in which individual differences dictated by reason sink into insignificance before the mass-reactions of the crowd." Certain instinctive reactions exhibit an absence of graduation according to conditions. They tend to occur in full strength. This form of reaction is known to physiologists under the title of "all-or-none." Dr. Rivers proposes to speak of instinctive behaviour as protopathic or epicritic according it is or is not subject to the "all-or-none" principle. Having discussed certain of the danger instincts, flight, aggression, collapse, &c., he proceeds to deal with the connection of instinct and suppression. He puts forth the fascinating hypothesis that suppression of instinctive reaction occurs when an animal adapted to one form of existence is forced by new needs to adopt new modes of reaction. He illustrates this by the examples of the life histories of the butterfly and the frog. And he also refers to what must have occurred in this way when man began arboreal existence. Having cleared the ground by this scientific preface, he goes on to the more practical applications of his theory.

We have to consider the meaning of the term "dissociation." "A suppressed experience does not remain passive, but acquires an independent activity of its own. The most characteristic example of dissociation is the fugue, in which a person shows behaviour, often of the most complicated kind, and lasting it may be for considerable periods, of which he is wholly unaware in the normal state. Dr. Rivers gives an example of such conduct. And he says: "If we accept the fugue as a typical and characteristic instance of dissociation, we are at once faced by another problem of definition. The subject of a fugue is certainly not unconscious. So far as we know, he is capable of experiencing all the modifications of consciousness which are open to the mind in its normal state. We have not at all to do with an example of the unconscious, but with consciousness cut off or dissociated from the consciousness of the normal waking life. . . All gradations" (of change of personality) "may be met with." These fugue states are, of course, quite well recognised by all authorities in morbid psychology. And, sooner or later, they will have to be taken account of by others. But they raise most difficult problems, especially in the medico-legal sphere. What is to be our reaction to criminal acts committed during such a state? How, in short, are we to deal with Jekyll for acts committed by Hyde? It would be hard to say that the subject of such a state did not "know the nature and quality of his act," or that he did not "know" that a certain act was "wrong." Yet these are the present legal criteria of "responsibility." These reflections indicate the necessity for a consideration of these legal dicta in the light of the fuller knowledge which we have acquired since the "McNaughten Judgment" in 1843. The question is still further complicated by the fact that, as Dr. Rivers says, "it is possible that during a fugue the normal personality may be independently conscious, and that the fugue-consciousness may persist beneath the surface in the normal state." Dr. Rivers does not, however, include "phobias," *i.e.*, morbid fears and impulses, under the head of fugues. And this exclusion should help to clear the air in the legal discussion which must take place some day.

Dr. Rivers points out that dissociation would be an almost necessary qualification for an animal which lives an amphibious existence. And as there is much reason to believe that man has, in his development, passed through an amphibious stage, the process of dissociation may be fitted into the biological scheme propounded in this book. We regard this chapter on dissociation as the most interesting in the whole book.

Dr. Rivers proceeds to discuss the concept of the "complex." And it is clear that a battle royal will arise as to the exact connotation of this word. Bernard Hart has defined a complex as "a system of connected ideas with a strong emotional tone and a tendency to produce actions of a certain definite character." Dr. Rivers, and the majority of authorities perhaps share his view, thinks that the term complex should be restricted to such a system only when it has been relegated to the unconscious. The correct use of the term will have to be settled, or the term given up, for nothing but confusion will arise if it is used in more than one sense. But we would plead that there is much to be said in favour of Hart's use of the term. It emphasises the essential unity of all mental life. Surely confusion can be avoided by speaking of a repressed, or suppressed, complex when we desire to bring out its unconscious character. Dr. Rivers thinks that pathology should have its own terms and concepts, although he admits that there is no hard-and-fast line between the healthy and the morbid.

The book contains interesting and helpful remarks upon the difficult problems of suggestion and hypnotism, problems which are, of course, closely linked together. And it goes on to the consideration of the "strangely neglected" problem of sleep, which bears much relation to both hypnotism and suggestion. "Going to sleep is an act which normally takes place unwittingly," and, in this respect, it closely resembles hypnotism. The process of dissociation is present in sleep; "this is specially obvious in sleep-walking, but the difference between it and the slight movement or utterance in a dream is one only in degree and not in kind." Dr. Rivers regards suppression as having a close relation to sleep, and he suggests that sleep is "an example of instinctive behaviour in which the process of suppression, originally subject to the all-or-none principle, has become capable of gradation in a high degree."

The author's main object is to suggest lines for thought, and directions in which future work should be carried on. He has admirably succeeded. The subjects of which he so ably treats are of the utmost importance to science and to society. The subject of this "Review of the Month" has just claims to the title of the "Book of the Year."

